# Validation of the Multiracial Youth Socialization Brief (MY‐Soc‐B) Scale with adolescents

**DOI:** 10.1111/jora.70121

**Published:** 2026-01-09

**Authors:** Annabelle L. Atkin, Nathan Lieng, Connor J. McNeil, N. Keita Christophe, Chelsea D. Williams

**Affiliations:** ^1^ Department of Human Development and Family Science Purdue University West Lafayette Indiana USA; ^2^ Department of Psychology McGill University Montreal Quebec Canada; ^3^ Department of Psychology Virginia Commonwealth University Richmond Virginia USA

**Keywords:** adolescent, Biracial, measurement, Multiracial, racial‐ethnic identity, racial‐ethnic socialization, validation

## Abstract

The present study validates a brief version of the Multiracial Youth Socialization Scale, the MY‐Soc‐B, which assesses Multiracial youths' experiences of receiving messages from their caregivers about race and culture. Using a sample of 318 diverse Multiracial American adolescents (*M*_age_ = 16.01; *SD* = 1.30), the original 62 items were reduced to 24 items via an examination of confirmatory factor analyses and conceptual clarity and fit of item content. The model fit strongly supported retaining the eight‐factor structure from the original full version. Validity and reliability of the eight subscales (i.e., navigating multiple heritages, Multiracial identity socialization, preparation for monoracism, negative socialization, race‐conscious socialization, diversity appreciation socialization, colorblind socialization, and silent socialization) were largely supported, with 20/24 correlations between the MY‐Soc‐B subscales and racial‐ethnic identity resolution, affirmation, and exploration replicating associations found with the original 62‐item MY‐Soc Scale.

## INTRODUCTION

Familial racial‐ethnic socialization (RES), the process whereby caregivers (i.e., parents or other family members raising a child) communicate about race, ethnicity, and culture with children, plays an important role in the development of youth (Hughes et al., 2006). In families with Multiracial American youth, who have racial heritage in multiple racial‐ethnic groups, socialization can impact how youth develop their racial‐ethnic identity (REI), learn to cope with discrimination, and navigate the world as racialized minorities (Atkin & Yoo, 2019). Research with monoracial minority groups has found that socialization can even mitigate or exacerbate associations between discrimination and mental health (Umaña‐Taylor & Hill, 2020), and thus socialization also has implications for youths' well‐being. However, there is very limited quantitative research that examines the correlates of familial RES among Multiracial adolescents.

While the dearth of socialization research with Multiracial adolescents is likely due to a number of factors, such as the relatively recent recognition of Multiracial individuals in U.S. society and the difficulty of recruiting Multiracial youth samples, one major factor is the limited measurement tools for studying Multiracial adolescents' experiences with RES (Atkin, Yoo, et al., 2022). Existing socialization measures assume a single‐race group membership, making them difficult for Multiracial participants with multiple group memberships to answer, and even more challenging for researchers to interpret their responses in terms of what racial group(s) participants had in mind when answering the question (Atkin, Yoo, et al., 2022). Moreover, these measures are not designed to capture specific socialization messages about Multiracial experiences (e.g., exclusion from one's own racial groups), which may play a significant role in Multiracial youth outcomes.

Having measures that assess RES experiences among Multiracial adolescents is critical for advancing the research on this important developmental process. The only existing peer‐reviewed measure specifically designed to assess familial RES of Multiracial youth, the Multiracial Youth Socialization (MY‐Soc) Scale (Atkin, Yoo, et al., 2022), was originally validated with a diverse sample of Multiracial emerging adults with a total of 8 subscales and 62 items. While this measure is comprehensive and allows for fine‐grained measurement of RES in Multiracial families, the long length of the scale may limit its practicality and uptake by researchers, as well as contribute to potential participant burden. The goal of the present study, therefore, is to create and validate a brief version of the MY‐Soc with a sample of Multiracial American adolescents to ensure that there is a short, practical, and psychometrically robust tool for assessing the unique socialization experiences of Multiracial adolescents.

### Theoretical framework

As was the case with the original validation study, the present study is guided by critical Multiracial theory, or MultiCrit (Harris, 2016), which highlights the unique positionality of Multiracial individuals within a monoracially structured society. The *monoracial paradigm* tenet, which acknowledges how society tries to force Multiracial people to fit within monoracial categories, informs our argument in the next section for why a distinct measure is needed to capture Multiracial socialization experiences beyond their monoracial component parts. The *experiential knowledge* tenet, which argues for centering the voices of Multiracial individuals, also informed our decision to validate the measure using Multiracial youths' reports instead of parent report. Furthermore, the *intersections of multiple racial identities* tenet helps us to acknowledge that Multiracial individuals are an incredibly diverse group, represented by individuals with many different combinations of racial heritages that uniquely inform their lived experiences.

Another important theory driving the study validation is Gonzales‐Backen's (2013) Conceptual Ecological Model of Biethnic Identity Formation, which posits that family ethnic socialization from various socialization agents (e.g., mother and father) plays an important role in REI exploration, resolution, and affirmation. Thus, our criterion‐related validity tests examine these three REI constructs as variables that would be associated with the MY‐Soc Brief constructs.

### Challenges of measuring RES in Multiracial populations

While commonly used among Multiracial samples, monoracial‐based measures of RES fail to capture the specific experiences of Multiracial individuals (Atkin, Yoo, et al., 2022). A major limitation of these measures being used with Multiracial populations is that certain items cannot sufficiently capture the existence of multiple racial‐ethnic backgrounds, cultures, and perspectives, as they presume a unified, monoracial outlook. Commonly seen items in socialization measures like “How often have your parents talked to you about important people or events in your group's history?” (Hughes & Johnson, 2001) can be confusing for Multiracial respondents because they may not know how to respond if their parents talked a lot about one of their groups but little about another; not to mention the frequency of messages can differ significantly across parents who come from different racial‐ethnic backgrounds (Atkin, Yoo, et al., 2022). As another example, an individual who identifies as a Black‐Asian Multiracial will have parents with unique perspectives and experiences with racial discrimination, which will in turn influence how they deliver preparation for bias messages.

Importantly, parents may not address the unique types of racial discrimination their children face due to being Multiracial, such as being excluded by their own racial in‐group peers (Atkin, Jackson, et al., 2022). Because of how previous socialization items have been designed with monoraciality implied, the measures may force the compartmentalization of a Multiracial individual's identity into monoracial categories, wherein individuals may be forced to “choose” an identity when answering the questions (Shaff et al., 2024). Though some studies may ask participants to answer items multiple times to capture each of their monoracial identities, this approach is still limited to a monoracial paradigm, instructing them to think of their identities as separate. Overall, monoracial‐based measures cannot adequately capture the unique experiences of Multiracial individuals and force this population to conform to monoracial paradigms (Atkin, Yoo, et al., 2022). Thus, the MY‐Soc was developed to assess the unique socialization experiences of Multiracial youth in navigating belonging to multiple racial groups and not fitting within society's monoracial paradigm.

### Review of RES literature with Multiracial adolescents

Within the current literature on adolescents' experiences of RES, our understanding of how RES impacts Multiracial adolescents specifically is lacking. The existing quantitative literature on Multiracial populations has largely focused on adults (e.g., Brittian et al., 2013; Christophe et al., 2021), and only three studies to our knowledge have focused on Multiracial adolescents' experiences with RES (González et al., 2006; Green et al., 2022; Winchester et al., 2023). González et al. (2006) examined how family race and gender composition were related to mean scores of familial RES and ethnic identity with a sample of Latine–White Biracial adolescents. However, they did not explore how RES and ethnic identity are associated with one another. Green and colleagues (2022) examined latent profiles of parental RES among Biracial Black–White adolescents with regard to the frequency and degree of similarity in parental messages, exploring these profiles' associations with factors such as racial pride and identity flexibility. Further, Winchester et al. (2023) explored the association between parental RES messages about Black identity among Biracial Black–White adolescents and parental characteristics, investigating relations to adolescents' choice of racial identification (e.g., exclusively Black, exclusively Biracial).

While these studies are innovative and have many strengths, the measures used were not designed to be used with the broader Multiracial population. For example, the González et al. (2006) study utilized the Familial Ethnic Socialization Measure (Umaña‐Taylor, 2001), which was designed with a monoracial perspective and did not specify which of the participants' identities the socialization messages pertained to. Moreover, this scale has not been validated with Multiracial adolescents. The other two studies (Green et al., 2022; Winchester et al., 2023) utilized the RSQ‐BA; Stokes (2021), which was designed to capture Black–White Biracial adolescents' experiences but is not meant for studying other Multiracial subgroups. This measure was also designed for a dissertation and has not been published in a peer‐reviewed journal, thereby limiting the accessibility and uptake of this measure. While this is a useful assessment for studying Black Biracial populations, it is equally important to have a reliable and valid measure that can capture the perspectives of the larger Multiracial adolescent population.

To address this gap in the literature, Atkin, Yoo, and colleagues (2022) developed the MY‐Soc Scale to capture socialization experiences of diverse Multiracial American emerging adults. The development of the MY‐Soc Scale was informed by a systematic review of the Multiracial socialization literature (Atkin & Yoo, 2019) and a qualitative investigation of Multiracial emerging adults' experiences with socialization (Atkin, Jackson, et al., 2022). The qualitative interviews and survey data for the scale validation for both the original and the present study were collected from Multiracial individuals in line with critical Multiracial theory (Harris, 2016), which advocates for centering the experiential knowledge of Multiracial individuals' voices in research concerning their experiences. The scale consists of 8 subscales. *Navigating Multiple Heritages Socialization* refers to whether caregivers taught them about their cultural backgrounds, as well as if they exposed them to people and cultural activities from their multiple racial‐ethnic groups. The second factor, *Multiracial Identity Socialization*, addresses how caregivers discussed their Multiracial identity or having multiple racial identities, and how to be proud of being Multiracial, whereas the third factor, *Preparation for Monoracism Socialization*, focuses on caregivers preparing them to deal with discrimination for being Multiracial. The fourth factor, *Negative Socialization*, refers to how caregivers expressed prejudicial attitudes and remarks toward one of the participants' racial‐ethnic groups and made them feel bad about their racial‐ethnic background. The fifth factor, *Race‐Conscious Socialization*, addresses how caregivers taught them about systemic racism and inequality. The sixth factor, *Colorblind Socialization*, refers to caregivers disregarding the significance of racism, while the seventh factor, *Diversity Appreciation Socialization*, addresses caregiver messages regarding the value of learning about and appreciating cultural differences, and accepting people from different racial‐ethnic backgrounds. Finally, the eighth factor, *Silent Socialization*, addresses caregivers' avoiding discussions of race or simply not talking about race. Silent socialization is distinct from colorblind socialization in that it represents parents who do not talk about race, while colorblind messages align with a colorblind ideology through explicit messages that deemphasize the significance of structural racism (Atkin, Jackson, et al., 2022; Neville et al., 2000). Thus, our conceptualization of colorblind socialization, based in the qualitative study and validation of the original measure published in 2022 (Atkin, Jackson, et al., 2022; Atkin, Yoo, et al., 2022), is overlapping but also differs from the Multiracial‐Black socialization model's conceptualization of color‐evasive socialization published in 2023 (Green & Bryant, 2023), which includes aspects distinctly captured by colorblind, silent, and diversity appreciation constructs in the MY‐Soc measure. These subscales range from 3 to 12 items each for a total of 62 items.

Another unique aspect of the scale's design is that it is set up to have adolescents report on their perception of socialization messages received from multiple caregivers (Atkin, Yoo, et al., 2022). Though the original validation study and the present study asked that participants choose up to two of their primary caregivers, defined to participants as “the people who had the most influence while raising you,” researchers could allow for more and/or specify which caregivers or other family members (e.g., siblings, grandparents) they would like the participants to consider when responding to the questions. The strength of this approach is that it more accurately allows researchers to capture the messages given by different socializing agents rather than asking participants to collectively rate their parents' socialization messages, which could easily differ. Giving participants the option to select who their caregivers are (e.g., stepfather, grandmother, two fathers, single mother) instead of assuming that two biological parents are raising them also accommodates the reality of diverse family formations that are becoming increasingly common.

### Current study

Studying socialization experiences with adolescents is crucial given that during this developmental period, Multiracial youth are living with their caregiver(s) and likely interacting more frequently, creating more opportunities for socialization. Moreover, a brief version of the MY‐Soc Scale is needed to relieve participant burden and increase feasibility for researchers to include the scale in their studies. Longer surveys are costly for researchers, and thus lengthy measures may be less likely to be included (Lavrakas, 2008). Moreover, survey length has been shown to be inversely related to data quality; thus, access to shorter scales may allow researchers to create shorter surveys and collect higher quality data (Galesic & Bosnjak, 2009). Thus, the present study sought to validate a brief version of the MY‐Soc Scale, the MY‐Soc‐B, with an adolescent sample.

We went through steps to reduce the number of items, conducted a confirmatory factor analysis (CFA) to test the measurement model and assess model fit, evaluated the reliability of the subscales using the alpha coefficient, and assessed validity through tests of associations with relevant constructs. Specifically, we sought to replicate the correlations in the original validation paper (Atkin, Yoo, et al., 2022) between the MY‐Soc subscales with the REI constructs of exploration, affirmation, and resolution, measured using items designed for Multiracial populations (Salahuddin & O'Brien, 2011; Yoo et al., 2016). Thus, we hypothesized that navigating multiple heritages socialization, Multiracial identity socialization, and diversity appreciation socialization would be positively correlated with exploration, affirmation, and resolution. In addition, we hypothesized that preparation for monoracism, race‐conscious socialization, and colorblind socialization would not be significantly associated with the REI components. Lastly, we hypothesized that negative socialization and silent socialization would only be negatively correlated with resolution, as was found in the original validation study.

## METHOD

### Participants

The present study included 318 Multiracial American adolescents between the ages of 14 and 18 years (*M*_age_ = 16.01; *SD* = 1.30). Participants self‐identified their gender as girl (51.6%), boy (46.5%), nonbinary/gender nonconforming (1.6%), and transgender boy (0.3%). Most participants were Biracial (80.0%), with the five largest Biracial groups in the sample being Black–White (*n* = 86, 27.0%), Latine–White (*n* = 81, 25.5%), Asian–White (*n* = 29, 9.1%), Native American–White (*n* = 18, 5.7%), and Black–Latine (*n* = 14, 4.4%). Among the participants, 46.9% were second‐generation (or beyond) Multiracial, meaning they have at least one Multiracial parent. See Table S2 for a more detailed breakdown of the racial backgrounds. The majority of the sample (94.3%) were born in the U.S., whereas 5.7% were born in another country. When prompted to select up to two primary caregivers from a list (“the people who had the most influence while raising you”), participants identified 11 different types of caregivers, with biological mothers and biological fathers being the most commonly reported caregiver types. Additionally, 15.1% of participants reported being raised by only one caregiver. A detailed breakdown of selected caregivers is reported in Table S2.

### Procedure

Participants were recruited using two methods: data collected directly by the first author's research lab, as well as through Qualtrics Panels, a participant recruitment service. Of the total sample of 318 participants, 36 were self‐collected by the researchers, and 282 were recruited through Qualtrics Panels. Participants were eligible if they were (a) Multiracial (i.e., having biological parents from two or more different racial backgrounds: Black, Asian, Pacific Islander, White, Latine, American Indian, and Middle Eastern/North African), (b) between the ages of 14 and 18, and (c) raised in the U.S. for most of their childhood. Participants completed a 20–30‐min online survey about themselves, their primary caregiver(s), and their experiences being Multiracial.

For the self‐collected data by the researchers, participants were recruited between October 2022 and January 2025 by sharing recruitment materials with (a) over 1000 high schools in major cities across the U.S. and local to the first author's university, (b) Multiracial student organizations across the U.S., (c) social media groups for Multiracial individuals and parents, and (d) contacts from the first author's research lab participant pool who indicated interest in being contacted for future studies. If interested, a primary caregiver of the Multiracial adolescent was required to provide consent for their child and complete an intake form, which the research team reviewed for eligibility before sending instructions to the adolescent on how to access, assent to, and complete the online survey. Participants were compensated with a $10 electronic gift card for their time spent completing the survey.

Qualtrics Panels collected data between December 2023 and March 2024. To prevent participants from falsely claiming a Multiracial identity to qualify for the study and receive compensation, a preliminary eligibility screening survey was used to determine whether participants or their children were eligible for the study based on child age and race. The specific details of the research study were provided only after participants reached the consent and assent portion of the survey. Participants could proceed if they met one of three scenarios: First, if participants indicated that they were 18 or older and a parent of a Multiracial child aged 14–18, they were informed about the consent process and instructed to provide consent themselves before passing the remainder of the survey to their adolescent child to complete. Second, if participants indicated they were 18 years of age, Multiracial, and not a parent, they were directed to the consent form where they could provide consent and complete the survey themselves. Lastly, if participants indicated that they were between the ages of 14 and 17 and Multiracial, they were directed to a section where one of their primary caregivers needed to provide consent before they could assent and complete the survey themselves. Participants who did not meet the eligibility criteria were automatically screened out of the survey via Qualtrics branch logic. Those who successfully completed the survey earned credits through Qualtrics Panels for their time, which could be redeemed for rewards.

### Measures

#### Multiracial Youth Socialization Scale

Parental Multiracial RES was measured using the full 62‐item MY‐Soc Scale (Atkin, Yoo, et al., 2022), which includes eight socialization subscales: navigating multiple heritages, Multiracial identity, preparation for monoracism, negative, colorblind, diversity appreciation, race‐conscious, and silent socialization. Instructions for the scale directed participants to “rate how much you agree that your parents/caregivers talked about or did what is described. This can be based on experiences you had growing up and/or your current experience” (see Supplemental Material A for detailed scale instructions, scale points, and items). Based on the specific caregiver roles indicated by the adolescent about their Caregiver 1 and Caregiver 2, the respective labels (e.g., biological mother) were piped into each survey item using Qualtrics logic. Participants rated each caregiver separately on the extent to which they engaged in the socialization content described in each item, using a 6‐point Likert‐type scale (1 = *strongly disagree* to 6 = *strongly agree*). This full version of the scale demonstrated reliability and validity with a sample of Multiracial emerging adults (Atkin, Yoo, et al., 2022).

#### Racial‐ethnic identity exploration

Exploration is a component of REI that involves participation in activities and content that help individuals think and learn more about their racial‐ethnic background (Umaña‐Taylor et al., 2004). Racial‐ethnic identity exploration was measured using the five‐item Multicultural Engagement subscale from the Multiracial Experiences Measure (Yoo et al., 2016). Participants rated how often they engaged in various culturally diverse events and experiences (e.g., “I celebrate holidays/celebrations of more than one culture”) on a 5‐point Likert‐type scale (1 = *almost never* to 5 = *almost always*). Item scores were averaged, with higher values indicating greater racial‐ethnic exploration. The internal consistency of the scale was acceptable (*α* = .79).

#### Racial‐ethnic identity affirmation

Affirmation is another component of REI that refers to the positive and/or negative feelings that one has toward their racial‐ethnic background (Umaña‐Taylor et al., 2004). Racial‐ethnic identity affirmation was measured using the five‐item Multiracial Pride subscale from the Multiracial Challenges and Resilience Scale (Salahuddin & O'Brien, 2011). Participants rated their agreement with statements reflecting their feelings about being Multiracial (e.g., “I am proud that I am Multiracial”) on a 6‐point Likert‐type scale (1 = *strongly disagree* to 6 = *strongly agree*). Item scores were averaged, with higher values reflecting greater racial‐ethnic affirmation. The internal consistency of the scale was acceptable (*α* = .79).

#### Racial‐ethnic identity resolution

Resolution is another facet of REI that describes the degree to which an individual has made sense of what their racial‐ethnic background means to them (Umaña‐Taylor et al., 2004). Racial‐ethnic identity resolution was measured using the five‐item Challenges with Racial Identity subscale from the Multiracial Challenges and Resilience Scale (Salahuddin & O'Brien, 2011). Participants rated their agreement with statements reflecting uncertainty or conflict about being Multiracial (e.g., “I feel as if I do NOT belong to any racial group”) on a 6‐point Likert‐type scale (1 = *strongly agree* to 6 = *strongly agree*). Items were reverse‐coded and averaged, such that higher values indicated greater racial‐ethnic resolution. The internal consistency of the scale was good (*α* = .83).

### Positionality statement

The first, third, fourth, and fifth author all identify as Multiracial, and the second author identifies as multiethnic Asian American. The Multiracial team members include individuals of Asian–White, Latine–White, and Black–White heritage. The first author's personal experiences struggling with how to respond to survey questions designed for monoracial populations and her interest in understanding the Multiracial socialization experiences of adolescents inspired this study. The authors' personal experiences, in combination with previous qualitative and quantitative research conducted by themselves and others, influenced the design, recruitment, item reduction, analysis, and writeup of the study. While there are strengths of the authors' positionality that improved many of these processes, there are also limitations given the differences in age between the authors and the adolescent participants and the incredible diversity among Multiracial backgrounds and experiences, most notably that multiple minority Multiracial individuals are not represented among the authorship team.

### Data analysis plan

#### MY‐Soc data preparation

Given that participants could endorse a Primary Caregiver 1 and Primary Caregiver 2^1^ from a range of roles, with biological mothers most often selected as Primary Caregiver 1 (81.1%) and biological fathers as Primary Caregiver 2 (52.2%), and because the MY‐Soc measure assesses RES messages from both caregivers (with each item having a response for Caregiver 1 and Caregiver 2), we randomly assigned half of the participants' scores to be from Caregiver 1 and the other half from Caregiver 2 to create a single score for each MY‐Soc item for analysis. Participants assigned to the Caregiver 2 group who reported being raised by a single primary caregiver did not have responses for the MY‐Soc Caregiver 2 items. Therefore, if they were chosen to provide a score from Caregiver 2, they were randomly swapped with participants in the Caregiver 1 group who had responses for both caregiver items, and their Caregiver 1 score was utilized given it was the only score available. This method helps ensure that the MY‐Soc measure is representative for caregivers diverse in genders, roles, and family contexts.

#### Item reduction

The goal for the item reduction process was to reduce each subscale to three items for a total of 24 items across the eight subscales to decrease response burden for adolescents while still allowing each construct to be modeled as a CFA. To determine which items to retain for the brief version of the MY‐Soc Scale, we first conducted a CFA with the adolescent sample using the original 62 items, and compared the resulting CFA loadings to those of the 62 items from the original validation paper's dataset with emerging adults (see Table 1). The subscale items with the three highest loadings from each dataset were noted. The preparation for monoracism subscale already had three items, so these items were retained without change. Any item that had one of the top three highest loadings for both datasets was retained for the brief version of the scale (*n* = 15). For the remainder of items that did not consistently have the highest loading across both datasets, the team engaged in discussions to determine which items to drop and retain, prioritizing items that had one of the top three highest loadings in at least one of the datasets. Items that were clear, easy to understand, and that strongly represented the construct were retained. In particular, appropriateness for developmental stage was considered by our team of developmental scientists to ensure that adolescents could comprehend and interpret the wording and content. Moreover, the original scale was developed with both emerging adults and adolescents in mind, as many of the items were informed by retrospective accounts of socialization experiences of participants prior to adulthood (Atkin, Yoo, et al., 2022). Moreover, the Flesch–Kincaid test determined the reading level of the items to be 61.3, on the low end of the range (60–70) for an eighth–ninth grade reading level. The score is likely inflated by the long, multi‐syllable words, “Multiracial” and “monoracial,” which are defined in the instructions to the scale for participants (see Supplemental Material A for instructions).

**TABLE 1:** Multiracial Youth Socialization (MY‐Soc) CFA factor loadings for emerging adult and adolescent samples.

Item	EA CFA loading	Ad CFA loading
*Factor 1: Navigating Multiple Heritages Socialization (7 items)*		
**1. Taught me customs specific to all of my different cultural backgrounds**	**0.753**	**0.720**
**2. Taught me about my family histories from all of my racial‐ethnic groups**	**0.690**	**0.772**
**3. Taught me about all of my racial‐ethnic backgrounds**	**0.777**	**0.713**
4. Exposed me to foods from all of my cultures	0.671	0.631
5. Had me participate in activities that taught me about my cultures	00.617	.617
6. Exposed me to other people in each of my racial‐ethnic communities	0.638	0.669
7. Exposed me to extended family members from all of my racial‐ethnic groups	0.585	0.583
*Factor 2: Multiracial Identity Socialization (10 items)*		
**8. Encouraged me to explore what it means to be Multiracial**	**0.761**	**0.764**
9. Told me that I can racially identify with any of my racial‐ethnic groups	0.507	0.651
10. Never talked to me about me being Multiracial (R)	0.681	0.559
11. Explained to me that I am Multiracial	0.576	0.674
12. Taught me multiple racial identity labels I could use	0.707	0.559
**13. Discussed our racial differences in positive ways**	**0.824**	**0.788**
**14. Taught me to be proud that I am Multiracial**	**0.564**	**0.757**
15. Told me to be proud of the way I look (e.g., skin color, hair color/type)	0.695	0.638
16. Told me that being Multiracial is special	0.678	0.619
17. Prepared me for others questioning me about my race	−0.626	0.677
*Factor 3: Preparation for Monoracism Socialization (3 items)*		
**18. Told me that monoracial people may not accept me as a member of their group**	**0.758**	**0.784**
**19. Told me that members of my racial groups may treat me differently because I am Multiracial**	**0.791**	**0.769**
**20. Told me that others may make me feel like I don't belong to my racial‐ethnic groups**	**0.843**	**0.773**
*Factor 4: Negative Socialization (12 items)*		
21. Did not pressure me to identify in any particular way (R)	−0.598	0.308
22. Laughs or makes jokes about my racial experiences without really addressing them	−0.541	0.748
**23. Said things that made me feel bad for not knowing enough about my culture**	**−0.740**	**0.733**
24. Said things that made me feel like I would be more attractive if I looked more like one of my monoracial groups	−0.625	0.739
**25. Said things that made me feel ashamed of being Multiracial**	**−0.668**	**0.763**
26. Said negative things (e.g., stereotypes, jokes, racist comments) about my other racial‐ethnic group(s)	−0.535	0.670
27. Expressed prejudicial attitudes toward my other racial‐ethnic group(s)	−0.662	0.717
28. Said I act too much like people from my other racial‐ethnic group(s)	−0.668	0.747
**29. Said things that made me feel like I do not belong to my (caregiver's) racial‐ethnic group**	**−0.579**	**0.829**
30. Said things that implied that my culture from my other racial‐ethnic group is bad or inferior	−0.563	0.738
31. Suggested that I should act more like a stereotype of my racial minority group(s)	0.509	0.676
21. Did not pressure me to identify in any particular way (R)	−0.591	0.655
*Factor 5: Race‐Conscious Socialization (7 items)*		
33. Taught me that there used to be laws that banned interracial marriage in the United States	0.614	−0.655
34. Encouraged me to participate in events or organizations working toward racial equality	0.643	−0.641
35. Taught me that people with lighter color skin have more privileges	0.492	−0.167
36. Made me aware of racial stereotypes affecting racial groups other than my own	0.628	−0.583
**37. Taught me that racism is reinforced by institutions in our society (e.g., legal system, schools, banks)**	**0.794**	**−0.474**
**38. Taught me about historical figures who fought for racial equality in America**	**0.725**	**−0.687**
**39. Taught me about unfair laws and policies in the United States that target racial‐ethnic minorities**	**0.821**	**−0.693**
*Factor 6: Colorblind Socialization (7 items)*		
**40. Says that they do not see race**	**0.544**	**0.542**
41. Says there are no racial differences between us	0.400	0.562
**42. Taught me that everyone has an equal opportunity for success regardless of their race**	**0.429**	**0.524**
43. Says there are more important things to worry about than race	0.612	0.496
44. Says that people are too sensitive about race	0.719	0.466
45. Says that racism is no longer an issue in the United States	0.710	0.508
**46. Says that White people also experience racism**	**0.671**	**0.553**
*Factor 7: Diversity Appreciation Socialization (10 items)*		
**47. Taught me that everyone's cultural differences make them unique**	**0.711**	**0.818**
48. Taught me to appreciate different cultures other than my own	0.786	0.745
49. Taught me to be respectful of people from different cultures	0.809	0.745
50. Taught me that cultures with different customs are not inferior	0.833	0.645
**51. Taught me to be accepting of people from all racial‐ethnic backgrounds**	**0.842**	**0.778**
52. Taught me to be open to cultural differences	0.810	0.770
**53. Taught me to not be judgmental of people from other cultures**	**0.823**	**0.798**
54. Taught me that the United States is enriched by its cultural diversity	0.696	0.615
55. Taught me not to judge or stereotype others based on their racial‐ethnic background	0.742	0.733
56. Encourages me to learn about other cultures other than my own	0.793	0.717
*Factor 8: Silent Socialization (6 items)*		
57. When I try to discuss race, my (caregiver) changes the subject	0.730	0.865
58. Never talks about race	0.753	0.687
**59. Avoids talking about race**	**0.876**	**0.815**
**60. Ignores the topic of race in conversation**	**0.849**	**0.782**
61. Does not know how to talk about race with me	0.741	0.801
**62. Uncomfortable talking about race**	**0.842**	**0.745**

*Note*: Responses given on a 6‐point scale of 1 (*strongly disagree*) to 6 (*strongly agree*). Items in boldface were retained for the MY‐Soc‐B.

Abbreviation: Ad, adolescent.

#### Short form model fit criteria

To test whether the eight‐factor structure found in the original emerging adult validation study (Atkin, Yoo, et al., 2022) replicated in this adolescent sample with the brief version of the scale, a CFA was conducted using Mplus with the 24 items. Model fit was evaluated using widely accepted criteria in structural equation modeling: a comparative fit index (CFI) >0.90, a root mean square error of approximation (RMSEA) <0.06, and a standardized root mean square residual (SRMR) <0.08 (Browne & Cudeck, 1993; Hu & Bentler, 1999). Research suggests that acceptable model fit is indicated when at least two of the three fit indices meet their recommended criteria (Merz et al., 2011; Mills et al., 2014). Furthermore, to test the dimensionality of the scale, we modeled three CFAs: a one‐factor model, an eight‐factor uncorrelated model, and an eight‐factor correlated model. The best‐fitting model was identified based on the previously mentioned fit indices, along with the model having the lowest Akaike Information Criterion (AIC).

#### Short form reliability and validity criteria

To evaluate internal reliability, Cronbach's alpha coefficients were computed and compared between the full MY‐Soc and MY‐Soc‐B subscales. The goal of the brief scale validation was to retain alpha coefficients similar to those of the full subscales, meeting standard conventions for acceptable internal consistency (*α* ≥ .60; Daud et al., 2018; Nunnally & Bernstein, 1994). Additionally, bivariate correlations between the full MY‐Soc and MY‐Soc‐B subscales were computed to assess shared variance, with the goal of identifying strong correlations as evidence of convergent validity between the full and brief forms.

Furthermore, to assess criterion‐related validity, additional bivariate correlations were computed between the brief MY‐Soc subscales and the theoretically related REI outcome variables. To replicate the original validation on emerging adults, which used the same REI variables (Atkin, Yoo, et al., 2022), and given the relatively large sample size of the current study, we interpreted significant correlations using both the effect size of the Pearson correlation coefficient (*r*) and the *p*‐value. We followed the effect size labeling criteria proposed by Funder and Ozer (2019): small (*r* = .10), medium (*r* = .20), and large (*r* = .30). While small effect sizes can be meaningful in this context, for the purposes of scale validation, we choose to only identify correlations equal to or exceeding a medium effect size (*r* ≥ .20) with a *p*‐value less than .05 as significant.

## RESULTS

### Development of the MY‐soc‐B

The item reduction process (see data analysis plan: item reduction for details) resulted in a reduction from 62 to 24 items, three for each of the eight subscales (see Table 2). All of the final items but one had one of the top three highest loadings for at least one of the datasets, and all of these loadings were above 0.54, well above the recommended minimum of 0.40 (Pett et al., 2003). The one item that was an exception had the fourth highest loading at 0.52 for the full scale adolescent CFA and was chosen for its conceptual fit with the other items in the brief subscale. This item, from the colorblind socialization scale, was chosen because it strongly represented the construct of colorblind socialization: “my [caregiver] taught me that everyone has an equal opportunity for success regardless of their race.”

**TABLE 2:** Multiracial Youth Socialization Brief (MY‐Soc‐B) Scale item descriptions and CFA factor loadings.

Item	CFA loading	*M*	*SD*
*Navigating Multiple Heritages Socialization*			
1. Taught me customs specific to all of my different cultural backgrounds	0.670	4.21	1.52
2. Taught me about my family histories from all of my racial‐ethnic groups	0.782	4.42	1.53
3. Taught me about all of my racial‐ethnic backgrounds	0.768	4.41	1.43
*Multiracial Identity Socialization*			
8. Encouraged me to explore what it means to be Multiracial	0.736	4.27	1.62
13. Discussed our racial differences in positive ways	0.812	4.77	1.39
14. Taught me to be proud that I am Multiracial	0.769	4.93	1.38
*Preparation for Monoracism Socialization*			
18. Told me that monoracial people may not accept me as a member of their group	0.805	3.13	1.80
19. Told me that members of my racial groups may treat me differently because I am Multiracial	0.758	3.59	1.80
20. Told me that others may make me feel like I don't belong to my racial‐ethnic groups	0.760	3.24	1.83
*Negative Socialization*			
23. Said things that made me feel bad for not knowing enough about my culture	0.677	1.97	1.40
25. Said things that made me feel ashamed of being Multiracial	0.763	1.75	1.33
29. Said things that made me feel like I do not belong to my (caregiver's) racial‐ethnic group	0.864	1.87	1.33
*Race‐Conscious Socialization*			
37. Taught me that racism is reinforced by institutions in our society (e.g., legal system, schools, banks)	0.518	3.83	1.71
38. Taught me about historical figures who fought for racial equality in America	0.654	4.37	1.60
39. Taught me about unfair laws and policies in the United States that target racial‐ethnic minorities	0.740	4.14	1.64
*Colorblind Socialization*			
40. Says that they do not see race	0.512	3.70	1.84
42. Taught me that everyone has an equal opportunity for success regardless of their race	0.643	4.22	1.69
46. Says that White people also experience racism	0.526	3.58	1.81
*Diversity Appreciation Socialization*			
47. Taught me that everyone's cultural differences make them unique	0.833	4.98	1.19
51. Taught me to be accepting of people from all racial‐ethnic backgrounds	0.755	5.23	1.15
53. Taught me to not be judgmental of people from other cultures	0.787	5.14	1.20
*Silent Socialization*			
59. Avoids talking about race	0.825	2.22	1.57
60. Ignores the topic of race in conversation	0.839	2.27	1.53
62. Uncomfortable talking about race	0.757	2.20	1.60

*Note*: Standardized CFA loadings presented; all factor loadings are significant at *p* < .001. Consistent with the original scale, responses are given on a 6‐point Likert scale of 1 (*strongly disagree*) to 6 (*strongly agree*).

Abbreviation: CFA, confirmatory factor analysis.

### Confirmatory factor analysis of the MY‐soc‐B

The model fit indices for the three CFA models for the MY‐Soc‐B are reported in Table 3. The correlated eight‐factor model with the navigating multiple heritages, Multiracial identity, preparation for monoracism, negative, colorblind, diversity appreciation, race‐conscious, and silent socialization subscales demonstrated the best fit, with all three of the fit indices (CFI, RMSEA, and SRMR) meeting criteria for acceptability. Table 2 displays the factor loadings for the correlated eight‐factor model. These findings provide evidence that, similar to the full MY‐Soc Scale, the MY‐Soc‐B is a multidimensional measure.

**TABLE 3:** Multiracial Youth Socialization Brief (MY‐Soc‐B) Scale model fit indices.

Model	*χ* ^2^	*df*	RMSEA [90% CI]	CFI	SRMR	AIC
1. Eight‐factor Correlated	405.71*	224	0.051 [0.043, 0.058]	0.93	0.06	24,750.67
2. Eight‐factor Uncorrelated	1127.94*	252	0.105 [0.098, 0.111]	0.67	0.23	25,513.58
3. One‐factor	1576.46*	252	0.129 [0.123, 0.135]	0.50	0.13	26,040.72

* *p*‐value < .001.

### Internal consistency

Table 4 presents the Cronbach's alpha coefficients for the full MY‐Soc and MY‐Soc‐B subscales. The MY‐Soc‐B subscales generally showed comparable coefficients to those of the full MY‐Soc. Seven of the MY‐Soc‐B subscale alpha coefficients demonstrated acceptable reliability, ranging from *α* = .67 to *α* = .85, while the colorblind socialization (*α* = .59) fell just below the .60 cutoff.

**TABLE 4:** Descriptive statistics and correlation matrix of study variables.

	1	2	3	4	5	6	7	8	9	10	11	12	13	14	15
1. NMHS_F	–														
2. NHMS_B	.92**	–													
3. MR Id_F	.76**	.68**	–												
4. MR Id_B	.72**	.66**	.91**	–											
5. PFM_F/B	.22**	.19**	.35**	.28**	–										
6. Neg_F	−.24**	−.22**	−.37**	−.34**	.19**	–									
7. Neg_B	−.23**	−.21**	−.39**	−.36**	.19**	.92**	–								
8. Race‐C_F	.53**	.49**	.59**	.55**	.36**	−.09	−.07	–							
9. Race‐C_B	.46**	.44**	.52**	.49**	.30**	−.14*	−.11	.91**	–						
10. Clrblnd_F	.17**	.17**	.16**	.13*	.09	.15*	.10	.06	.05	–					
11. Clrblnd_B	.23**	.24**	.29**	.27**	.11	−.08	−.11*	.12*	.12*	.85**	–				
12. DA_F	.56**	.51**	.65**	.64**	.08	−.49**	−.44**	.60**	.54**	.27**	.40**	–			
13. DA_B	.48**	.44**	.61**	.60**	.08	−.46**	−.44**	.53**	.48**	.25**	.37**	.93**	–		
14. Silent_F	−.34**	−.34**	−.48**	−.49**	.03	.65**	.64**	−.24**	−.26**	.28**	.06	−.42**	−.42**	–	
15. Silent_B	−.28**	−.27**	−.40**	−.42**	.02	.57**	.56**	−.21**	−.23**	.31**	.12*	−.34**	−.34**	.95**	–
*α*	.85	.78	.89	.81	.82	.91	.79	.76	.67	.73	.59	.92	.85	.90	.84
*M*	4.39	4.36	4.57	4.66	3.31	1.88	1.84	4.06	4.11	3.49	3.82	5.03	5.13	2.18	2.22
*SD*	1.08	1.24	1.05	1.25	1.55	0.98	1.12	1.04	1.28	1.06	1.32	0.96	1.02	1.26	1.37
REI‐Exp	.42**	.38**	.24**	.25**	.04	−.05	−.05	.33**	.33**	−.00	.05	.26**	.19**	−.15*	−.12*
REI‐Aff	.46**	.37**	.49**	.50**	.19**	−.15*	−.17**	.31**	.33**	.09	.17**	.33**	.25**	−.25**	−.19**
REI‐Res	.35**	.32**	.37**	.34**	−.01	−.39**	−.38**	.14*	.16**	.09	.20**	.29**	.25**	−.36**	−.29**

Abbreviations: B, brief version; Clrblnd, colorblind socialization; DA, diversity appreciation; F, full version; MR Id, Multiracial identity socialization; Neg, negative socialization; NHMS, navigating multiple heritage socialization; PFM, preparation for monoracism (same items for both full and brief version); Race‐C, race‐conscious socialization.

* *p* < .05;

** *p* < .01.

### Convergent and criterion‐related validity

Bivariate correlations were computed among the full MY‐Soc subscales, the MY‐Soc‐B subscales, and the REI variables to assess convergent validity between the full and brief versions, as well as criterion‐related validity in relation to theoretically relevant REI variables (see Table 4). Only correlations equal to or exceeding medium effect sizes (i.e., *r* ≥ .20) that had a *p*‐value less than .05 were interpreted as significant. Correlations between the full MY‐Soc and MY‐Soc‐B subscales ranged from *r* = .84 (colorblind socialization) to *r* = .95 (silent socialization), supporting convergent validity and indicating strong conceptual retention in the brief subscales. The brief subscales demonstrated correlation values comparable to those of the full subscales, with associations generally aligning with hypothesized directions for the REI outcome variables, though four exceptions among the 24 hypothesized associations are noted below.

As hypothesized, the brief versions of the navigating multiple heritages, Multiracial identity, and diversity appreciation socialization subscales were positively correlated with REI exploration, affirmation, and resolution, except that the brief diversity appreciation subscale was not significantly correlated with REI exploration (although it approached significance; *r* = .19; *p* = .002). Effect sizes for these brief subscales ranged from medium (*r* = .25 for the correlations between diversity appreciation and both REI affirmation and resolution) to large (*r* = .50 for the correlation between multiracial identity socialization and REI affirmation).

Partially supporting our hypothesis that these brief subscales would show no significant correlations with the REI outcome variables, the preparation for monoracism subscale was not correlated with any REI variables. In contrast, the race‐conscious socialization subscale was positively correlated with REI exploration and affirmation, and the colorblind socialization subscale was correlated with REI resolution. The brief subscales showed medium‐to‐large effect sizes with the REI outcome variables, ranging from *r* = .20 for the correlation between colorblind socialization and REI resolution to *r* = .33 for the correlations between race‐conscious socialization and both REI exploration and affirmation.

Finally, in support of our hypothesis that negative socialization and silent socialization would be negatively associated solely with REI resolution, both negative socialization and silent socialization were negatively correlated with only REI resolution. Effect sizes for these brief subscales ranged from medium (*r* = −.29 for the association between silent socialization and REI resolution) to large (*r* = −.38 for the association between negative socialization and REI resolution).

Overall, the significant associations largely replicate those found in the original validation study with emerging adults and follow the expected directions based on each socialization message type's conceptual influence on REI. These findings suggest that even after trimming items, the MY‐Soc‐B subscales show convergent and criterion‐related validity that closely matches the full version.

## DISCUSSION

The present study sought to validate a brief version of the MY‐Soc Scale (Atkin, Yoo, et al., 2022) with a Multiracial American adolescent sample. The original MY‐Soc Scale is the only validated and published scale for assessing familial socialization experiences specific to Multiracial youth (i.e., navigating multiple heritages, Multiracial identity socialization, preparation for monoracism, negative socialization), as well as distinct types of socialization related to different connotations of egalitarian messages (i.e., race‐conscious, diversity appreciation, and colorblind socialization), and an explicit measure of silent socialization. Given the significance of socialization processes in youth development, it is critical to have measures to capture these experiences among the fastest growing racial group in the U.S., Multiracial Americans. Having a brief version of the scale is useful given that the shorter length is more practical and efficient for research and applied settings, potentially improving participant response rates and reducing participant burden and missingness in future studies. To validate the MY‐Soc‐B, we engaged in item reduction and conducted validity and reliability tests with the brief version of the scale.

The brief measure replicated the eight‐subscale structure of the original 62 item measure, reducing each subscale to 3 items, for a total of 24 items for the MY‐Soc‐B. CFA loadings and model comparisons confirmed that the same eight‐factor correlated model structure as the original model had a strong fit. Poor fit for the one‐factor model suggests that subscales should not be combined to create one overall MY‐Soc Scale for analyses, as each subscale represents a distinct conceptual dimension.

Reliability tests found acceptable fit for seven of the eight subscales, and poor fit for the colorblind socialization subscale. Although the brief colorblind socialization subscale yielded a Cronbach's alpha coefficient of *α* = .59, Taber (2018) argues that additional context should be considered when assessing the reliability of a scale. The equation for calculating alpha penalizes measures with fewer items and incentivizes longer measures and measures with redundant items; thus, a higher alpha coefficient for a scale with more items may reflect redundancy rather than true reliability (Bäcker et al., 2024). Therefore, our lower alpha may be due to our lower number of items. However, despite the small number of items, the three items thoroughly cover the construct of colorblind ideology, including the distinct but related aspects of color evasion (i.e., “Says that they don't see race”) and power evasion (i.e., “Says that White people also experience racism”; Mekawi et al., 2020). Additionally, the 95% confidence interval [*α* = .52, .67] suggests that the true alpha coefficient may fall within an acceptable range. Given the relatively lower reliability for the brief colorblind socialization subscale, researchers might consider including the full version of the subscale, as further testing in other samples may be needed to establish greater confidence in the subscales' reliability.

Convergent validity was established through associations with large effect sizes between the full and brief versions of each MY‐Soc subscale, suggesting that the brief version assesses similar constructs as the original. Criterion‐related validity was established through the hypothesized associations with medium‐to‐large effect sizes between the MY‐Soc‐B subscales and the REI subscales. As hypothesized, the brief versions of the navigating multiple heritages socialization, Multiracial identity socialization, and diversity appreciation socialization subscales were all positively correlated with REI exploration, affirmation, and resolution at a medium or large effect size, with the exception of the association between diversity appreciation socialization and REI exploration, which was just below the cutoff for medium effect size. One possible explanation for this is that because diversity appreciation is more focused on teaching youth to be accepting of other people's cultures, this is not as relevant for their engagement with their own cultures. In the original validation paper, the only association among these subscales that did not reach significance at a medium effect size was the one between diversity appreciation socialization and REI resolution (Atkin, Yoo, et al., 2022).

A recent meta‐analysis by Huguley et al. (2019) indicated that of 68 studies across all racial groups, there was a medium effect size for the association between parental pride and heritage messages with REI. However, only one of the published studies from the review focused on a Biracial sample; among Latino–White and Asian–White college students, ethnic socialization was positively related to REI exploration and resolution, but not affirmation (Brittian et al., 2013). However, all of the measures used were designed for monoracial populations and did not specify an ethnic group for the participants to think about when answering the questions. One qualitative interview study with Multiracial and bisexual female college students found that in their reflections on adolescence, they indicated how the importance of having family to talk with about their racial heritage and encouragement to explore their racial heritage (i.e., navigating multiple heritages socialization) contributed to their understanding of their racial identities (King, 2013). Moreover, in another interview study, Multiracial‐Black college students reported that having parents talk with them about their Multiracial identity shaped their racial identity development (Jones & Rogers, 2023). Qualitative Multiracial socialization literature suggests that distinguishing cultural socialization and identity socialization is critical because the cultures that youth are socialized about may not always align with the identities they are socialized about or that are important to the child (Atkin & Jackson, 2021; Atkin, Jackson, et al., 2022). Thus, these three subscales capture more breadth and nuance in regard to the relevant messages that may promote REI development for Multiracial youth. In sum, our findings suggest that receiving socialization messages that teach youth to explore their multiple heritages, have awareness of their Multiracial identity, and appreciate diversity is likely related to a strong REI for Multiracial adolescents, though further research is needed to examine whether teaching youth to appreciate diversity of all groups is associated with exploration of their own identity.

Preparation for monoracism was not significantly associated with any of the REI constructs, similar to the original study. Huguley et al.'s (2019) meta‐analysis reported mixed findings and a very small effect size in the association between bias messages and REI. Given that the preparation for monoracism messages in the MY‐Soc‐B focus on preparation for rejection from groups the adolescent belongs to and the current study's measure of REI was focused on Multiracial identity, it will be interesting to explore associations with different REI options in future studies. For instance, one question might be whether Multiracial youth who receive a lot of preparation for monoracism messages are more likely to identify with a Multiracial or monoracial label.

In partial support of the hypothesis, race‐conscious socialization was not significantly associated with resolution. However, while not associated with any REI constructs in the original validation study, race‐conscious socialization was positively associated with both REI exploration and affirmation at a medium effect size (Atkin, Yoo, et al., 2022). In other words, Multiracial adolescents who reported receiving messages that taught about systemic racism in the U.S. were more likely to have engaged in activities to explore their cultures and feel positively about being Multiracial. This is an interesting finding given that raising awareness about how systems are working against people of color could potentially lead youth to not want to associate with their marginalized cultures or Multiracial identity. An interview study with Black–White Multiracial adolescents suggests that they often receive messages from peers and others outside their family that reinforce White supremacy by invalidating their racial‐ethnic identities, and they have to negotiate their position within the racial hierarchy as they develop their identity (Jones & Rogers, 2022). It will be important for future research to explore how parents raising awareness of systemic racism and White supremacy influences REI development for Multiracial youth.

Also in partial support of the hypothesis, colorblind socialization was not significantly associated with exploration or affirmation. However, colorblind socialization was positively associated with RES resolution in the present study at a medium effect size despite not being associated with any REI constructs in the original validation study. Thus, Multiracial adolescents who reported receiving messages that taught them that race is not consequential for people of color were more likely to have an understanding of what their REI means to them. This finding is particularly surprising given that youth received messages devaluing race, and yet they personally felt that their REI was meaningful. Future studies are needed to further explore whether this association holds. The only other study to our knowledge to examine correlations using a similar construct in the family context with REI among Multiracial individuals was a dissertation with a sample of Black–White Biracial adolescents, wherein color‐evasive socialization was found to be positively correlated with Black private regard and Multiracial pride (Stokes, 2021).

Lastly, as we hypothesized, negative and silent socialization were only negatively correlated with resolution, both at a medium effect size. In other words, having caregivers who provided messages that invalidated youths' identity or negatively portrayed one or more of their racial groups or who were silent on the topic of race was associated with Multiracial adolescents not being as confident about what their REI means to them and having more challenges with their Multiracial identity. Though there is little previous research that has examined negative socialization or explicitly measured silent socialization, these findings are in line with previous research suggesting that low scores on RES, which could be interpreted as little or no (i.e., silent) socialization, are often associated with lower scores for REI (Huguley et al., 2019). Findings also support previous qualitative research suggesting that Multiracial females who had a lack of race discussions (i.e., silent socialization) or received negative messages (i.e., negative socialization) at home consequently lacked awareness or ownership of their identities (King, 2013).

Though no studies to our knowledge (besides the original validation study) have reported correlations between RES and REI with a Multiracial sample, studies examining profiles of combinations of RES messages have found that profiles characterized by higher frequencies of RES are broadly related to stronger REI for Multiracial college students (Christophe et al., 2024) and Multiracial‐Black adolescents (Green et al., 2022). We hope that the MY‐Soc‐B can be useful for future studies examining socialization messages both separately and in combination to further advance the literature.

### Recommendations for use of the MY‐Soc‐B

The full version of the MY‐Soc was validated with a sample of Multiracial American emerging adults, and this article validates the MY‐Soc‐B with a sample of Multiracial American adolescents. Future studies could validate the MY‐Soc‐B with Multiracial American adults within and beyond emerging adulthood. The first author has also reworded the scale items for use with caregivers to capture their perspective of the socialization messages they provide their Multiracial child. Though the caregiver version has not been validated due to small sample sizes, items are available upon request, and future validation of the scale from the perspective of other socializing agents (e.g., parents, grandparents, teachers) is another next step for advancing the literature to understand the role other socializing agents play and the discrepancies between parent and child report of socialization.

Notably, the original and brief version of this scale were created to ensure that Multiracial youths' experiences of socialization could be captured in research. There may be research questions for which understanding experiences of socialization for one of the monoracial groups a Multiracial individual belongs to would be of interest, and in this case, other existing RES scales could be used. For instance, if a researcher wanted to know whether parents were preparing Black Biracial youth for discrimination based on their Black heritage, they might use Hughes and Johnson' (2001) preparation for bias subscale and specify to the participant that they are asking about their socialization experiences associated with being Black. In their study, Christophe and colleagues (2021) asked Multiracial emerging adults how each of their parents socialized them about each parent's racial group using Hughes and Johnson' (2001) parental racial socialization scale. While these experiences are important to study, as noted earlier, there is more to Multiracial youths' RES experiences than communicating with socializing agents about their monoracial groups. Thus, it is recommended that researchers carefully consider the unique racialized experiences of Multiracial youth when developing their research questions, and consider using a scale that captures these experiences, such as the MY‐Soc or MY‐Soc‐B. The Racial Socialization Questionnaire for Biracial Adolescents (RSQ‐BA) (Stokes, 2021) is also an option if researchers are specifically studying Black Biracial youth, and Green et al. (2022) provide the first example to our knowledge of asking about RES messages for both a monoracial (Black) identity and Biracial identity using this measure.

Another important point to note is that while four of the subscales specifically focus on uniquely Multiracial experiences (i.e., navigating multiple heritages, Multiracial identity socialization, preparation for monoracism, negative socialization), the other four (i.e., race‐conscious socialization, colorblind socialization, diversity appreciation socialization, and silent socialization) work both for Multiracial youth and monoracial youth of all races. The items for the latter four subscales were developed based on interviews with Multiracial emerging adults (Atkin, Yoo, et al., 2022), but are general enough to be relevant for all racial groups, especially in the MY‐Soc‐B.^2^ Moreover, these subscales fill a gap in the literature, seeking to distinguish the different connotations that egalitarian socialization messages, or messages about treating everyone equally, could have (for more details, see Atkin, Jackson, et al., 2022). These four subscales from the original MY‐Soc validation study have already been used with a sample of Asian Americans (Atkin & Ahn, 2022) and can collectively advance the literature regarding how socializing agents talk about systemic racism and cultural diversity with their children, or promote colorblind racial ideology or avoid discussing race with their children altogether.

### Limitations and future directions

While one strength of the MY‐Soc‐B is its broad applicability across diverse Multiracial groups, as noted earlier, it does only focus on Multiracial socialization experiences, though Multiracial youth also likely receive messages about specific monoracial groups they are members of. For instance, this measure assesses preparation for monoracism, but does not assess preparation for discrimination or bias specific to monoracial groups that the child might experience (e.g., a Biracial Asian child's parents discussing the model minority myth, a stereotype relevant for Asian individuals). But as noted above, other existing measures could be included in addition to the MY‐Soc to capture monoracial‐specific socialization messages.

Future research should seek to understand both the aggregate and disaggregated experiences of specific Multiracial subgroups to understand the similarities and differences faced by youth with different racial backgrounds. Other factors that might influence the socialization messages they receive (e.g., phenotype, race of the socializing agent, composition of school and community contexts, and family structure) and associated constructs (e.g., mental health, critical consciousness) should also be explored. As previously mentioned, future research should also ask about other socializing agents besides the two primary caregivers (e.g., siblings, grandparents), and collect data from the perspectives of the socializing agents to examine discrepancies with youth report and determine whether the report of the person giving or receiving the message is more predictive of various outcomes. Finally, daily diary and longitudinal studies will be important to understand how socialization messages are experienced over time and in relation to other developmental processes.

### Conclusion

In sum, this study provides validation for a 24‐item brief socialization scale for Multiracial American adolescents, the MY‐Soc‐B. It is also the first study to examine correlations between RES and REI among a racially diverse Multiracial adolescent sample. Despite the large body of literature that suggests RES has strong associations with REI dimensions (Huguley et al., 2019) and mental health outcomes (Hughes et al., 2006; Umaña‐Taylor & Hill, 2020) among monoracial youth, there has not yet been a study that has examined these associations among a racially diverse sample of Multiracial adolescents using a measure that captures socialization about uniquely Multiracial experiences. With the MY‐Soc‐B available, future studies can now advance the literature to understand the role that Multiracial youth socialization plays in developmental processes.

## AUTHOR CONTRIBUTIONS

**Annabelle L. Atkin:** Conceptualization; investigation; funding acquisition; writing – original draft; writing – review and editing; methodology; visualization; formal analysis; project administration; data curation; supervision; resources. **Nathan Lieng:** Data curation; writing – original draft; visualization; writing – review and editing; investigation; formal analysis. **Connor J. McNeil:** Writing – original draft. **N. Keita Christophe:** Writing – review and editing; conceptualization. **Chelsea D. Williams:** Writing – review and editing; conceptualization; formal analysis.

## FUNDING INFORMATION

The authors would like to thank the Arizona State University Institute for Social Science Research for funding this study.

## CONFLICT OF INTEREST STATEMENT

The authors do not have any conflicts of interest to declare.

## ETHICS STATEMENT

This study was approved by Purdue IRB on 5/3/2022, reference #IRB‐2021‐1465.

## CONSENT STATEMENT

Parents provided consent if the Multiracial adolescent was between the ages 14 and 17, and then the adolescent provided assent. If participants indicated they were 18 years of age, they provided consent themselves.

## Supporting information


Data S1:

## Data Availability

The data that support the findings of this study are available from the corresponding author upon reasonable request.

## Footnotes

^1^
Primary Caregiver 1 and primary Caregiver 2 do not indicate any ranked ordering but rather the order in which participants listed their primary caregivers.

^2^
In the full version of the MY‐Soc, all items could be used with other racial groups except that the items “Taught me that there used to be laws that banned interracial marriage in the United States” from the race‐conscious socialization scale and “Says there are no racial differences between us” from the colorblind socialization subscale are more relevant to Multiracial youth. Note that neither item was retained for the MY‐Soc‐B.
