# Supplementary material for: Validation of the Multiracial Youth Socialization Brief (MY‐Soc‐B) Scale with adolescents
Source: J Res Adolesc. 2026 Jan 9;36(1):e70121. doi: 10.1111/jora.70121 (PMC12789715; doi:10.1111/jora.70121)
Supplement: Supplementary file 1 — Data S1: [file JORA-36-0-s001.docx]

Supplemental Table 1.

*Racial breakdown of participants based on race reported for their biological parents (N=318)*

| Racial Background | Frequency |
| --- | --- |
| Black-White | 86 (27.0%) |
| Latinx-White | 81 (25.5%) |
| Asian-White | 29 (9.1%) |
| Native American-White | 18 (5.7%) |
| Black-Latinx | 14 (4.4%) |
| Black-Asian | 5 (1.6%) |
| Asian-Latinx | 5 (1.6%) |
| MENA-White | 3 (0.9%) |
| NHPI-White | 2 (0.6%) |
| Black-Native American | 2 (0.6%) |
| Asian-MENA | 1 (0.3%) |
| Native American-Latinx | 1 (0.3%) |
| POC multiple minorities | 1 (0.3%) |
| White multiple minorities | 69 (21.7%) |
| Second-generation Multiracial | 149 (46.9%) |
| Not listed | 1 (0.3%) |

*Note*. MENA = Middle Eastern and North African, NHPI = Native Hawaiian and Pacific Islander, POC = People of Color.

Supplemental Table 2.

*Report of Participants’ Primary Caregivers*

| Label | Caregiver 1 | Caregiver 2 |
| --- | --- | --- |
| Biological mother | 258 (81.1%) | 43 (13.5%) |
| Biological father | 47 (14.8%) | 166 (52.2%) |
| Adoptive mother | 5 (1.6%) | 3 (0.9%) |
| Adoptive father | 1 (0.3%) | 4 (1.3%) |
| Stepmother | -- | 6 (1.9%) |
| Stepfather | -- | 26 (8.2%) |
| Grandma | 6 (1.9%) | 14 (4.4%) |
| Grandpa | 1 (0.3%) | 3 (0.9%) |
| Second mother | - | 1 (0.3%) |
| Uncle | - | 1 (0.3%) |
| Brother | - | 1 (0.3%) |
| Raised by only one primary caregiver | - | 48 (15.1%) |
| Not specified | - | 2 (0.6%) |

Supplemental Material A

**Instructions to Researchers**

**Notes on setting up the scale:**

1. By allowing participants to select two primary caregivers from a list in the beginning of the survey, the caregivers they chose can be populated as the title of two response scale columns when they respond to the measure items (e.g., biological mother in one column, stepfather in the other column). This allows participants to indicate the level of agreement that they received the message described by each caregiver separately.
2. Items should be presented to participants in a randomized order.

**Disclaimers to include before Multiracial Youth Socialization Scale:**

Please read and acknowledge the following:

1. In this study, we use**"Multiracial"**to refer to people of any mixed race background, including biracial, as long as they have biological parents from two or more of the following groups: White, Asian, Black, Latine, Pacific Islander, American Indian, or Middle Eastern/North African. The term **"monoracial"**refers to people from only one of the listed racial groups.

- I understand

1. These questions ask about whether your parents/caregivers **explicitly**said or did something. Disagreeing with these questions does not mean that your parents/caregivers did not think these things were important or that they did the opposite of what is described, but simply that you do not recall them intentionally engaging in these conversations or actions.
   - I understand
2. The following questions ask you to rate **how much you agree that your parents/caregivers talked about or did what is described**. This can be based on**experiences you had growing up and/or your current experience.**Please answer the following questions about the messages you received**from each of your primary caregivers by filling in the blank with the person listed at the top of the column.**If you listed early on in the survey that you only have one primary caregiver, you may leave the second column blank.
   - I understand

**Multiracial Youth Socialization – Brief (MY-Soc-B) Scale (24 items)**

| Strongly Disagree | Disagree | Slightly Disagree | Slightly Agree | Agree | Strongly Agree |
| --- | --- | --- | --- | --- | --- |

**Directions:**The following questions ask you to rate **how much you agree that your parents/caregivers talked about or did what is described**. This can be based on**experiences you had growing up and/or your current experience.**

Please answer the following questions about the messages you received**from each of your primary caregivers by filling in the blank with the person listed at the top of the column.**If you listed early on in the survey that you only have one primary caregiver, you may leave the second column blank.

Navigating Multiple Heritages Socialization

1. My (caregiver) taught me customs specific to all of my different cultural backgrounds
2. My (caregiver) taught me about my family histories from all of my racial-ethnic groups
3. My (caregiver) taught me about all of my racial-ethnic heritage(s)

Multiracial Identity Socialization

1. My (caregiver) encouraged me to explore what it means to be Multiracial
2. My (caregiver) discussed our racial differences in positive ways
3. My (caregiver) taught me to be proud that I am Multiracial

Preparation for Monoracism Socialization

1. My (caregiver) told me that monoracial people may not accept me as a member of their group
2. My (caregiver) told me that members of my racial groups may treat me differently because I am Multiracial
3. My (caregiver) told me that others may make me feel like I don't belong to my racial-ethnic groups

Negative Socialization

1. My (caregiver) said things that made me feel bad for not knowing enough about my culture
2. My (caregiver) said things that made me feel ashamed of being Multiracial
3. My (caregiver) said things that made me feel like I do not belong to my (caregiver's) racial-ethnic group

Race-conscious Socialization

1. My (caregiver) taught me that racism is reinforced by institutions in our society (e.g., legal system, schools, banks)
2. My (caregiver) taught me about historical figures who fought for racial equality in America
3. My (caregiver) taught me about unfair laws and policies in the United States that target racial-ethnic minorities

Colorblind Socialization

1. My (caregiver) says that they don't see race
2. My (caregiver) taught me that everyone has an equal opportunity for success regardless of their race
3. My (caregiver) says that White people also experience racism

Diversity Appreciation Socialization

1. My (caregiver) taught me that everyone's cultural differences make them unique
2. My (caregiver) taught me to be accepting of people from all racial-ethnic backgrounds
3. My (caregiver) taught me to **not** be judgmental of people from other cultures

Silent Socialization

1. My (caregiver) avoids talking about race
2. My (caregiver) ignores the topic of race in conversation
3. My (caregiver) is uncomfortable talking about race
